# Transcription factor mesenchyme homeobox protein 2 (MEOX2) modulates nociceptor function

**DOI:** 10.1111/febs.16347

**Published:** 2022-02-16

**Authors:** Tomislav Kokotović, Ewelina M. Lenartowicz, Michiel Langeslag, Cosmin I. Ciotu, Christopher W. Fell, Angelica Scaramuzza, Michael J. M. Fischer, Michaela Kress, Josef M. Penninger, Vanja Nagy

**Affiliations:** ^1^ 27271 Ludwig Boltzmann Institute for Rare and Undiagnosed Diseases Vienna Austria; ^2^ CeMM‐Research Center for Molecular Medicine of the Austrian Academy of Sciences Vienna Austria; ^3^ 27271 Department of Neurology Medical University of Vienna Austria; ^4^ 27280 Department of Physiology and Medical Physics Institute of Physiology Medical University of Innsbruck Austria; ^5^ 27255 Institute of Pharmacy and Center for Molecular Biosciences Innsbruck (CMBI) University of Innsbruck Austria; ^6^ 27280 Department of Pharmacology Medical University of Innsbruck Austria; ^7^ 27271 Institute of Physiology Medical University of Vienna Austria; ^8^ Institute of Molecular Biotechnology of the Austrian Academy of Sciences VBC – Vienna BioCenter Campus Vienna Austria; ^9^ Department of Medical Genetics Life Science Institute University of British Columbia Vancouver Canada

**Keywords:** dorsal root ganglia, *MEOX2*, *Nav1.8*, nociception, pain

## Abstract

Mesenchyme homeobox protein 2 (MEOX2) is a transcription factor involved in mesoderm differentiation, including development of bones, muscles, vasculature and dermatomes. We have previously identified dysregulation of *MEOX2* in fibroblasts from Congenital Insensitivity to Pain patients, and confirmed that *btn*, the *Drosophila* homologue of *MEOX2*, plays a role in nocifensive responses to noxious heat stimuli. To determine the importance of MEOX2 in the mammalian peripheral nervous system, we used a *Meox2* heterozygous (*Meox2* ^+/−^) mouse model to characterise its function in the sensory nervous system, and more specifically, in nociception. MEOX2 is expressed in the mouse dorsal root ganglia (DRG) and spinal cord, and localises in the nuclei of a subset of sensory neurons. Functional studies of the mouse model, including behavioural, cellular and electrophysiological analyses, showed altered nociception encompassing impaired action potential initiation upon depolarisation. Mechanistically, we noted decreased expression of *Scn9a* and *Scn11a* genes encoding Na_v_1.7 and Na_v_1.9 voltage‐gated sodium channels respectively, that are crucial in subthreshold amplification and action potential initiation in nociceptors. Further transcriptomic analyses of *Meox2* ^+/−^ DRG revealed downregulation of a specific subset of genes including those previously associated with pain perception, such as *PENK* and *NPY*. Based on these observations, we propose a novel role of MEOX2 in primary afferent nociceptor neurons for the maintenance of a transcriptional programme required for proper perception of acute and inflammatory noxious stimuli.

## Graphical abstract

Mesenchyme homeobox protein 2 (MEOX2) is a transcription factor important for the development of bones and muscles. We report that MEOX2 is expressed in the nervous system, particularly localised to nociceptive dorsal root ganglia neurons, responsible for pain detection. Our multidisciplinary study of haploinsufficient MEOX2 mouse model revealed that MEOX2 is responsible for fine‐tuning nociceptor action potential generation and behavioural responses to noxious stimuli by regulation of pain gene expression, including particular sodium channel subunits.

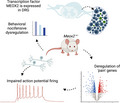

**Abbreviations**
*Actn3*
: gene, Actinin alpha 3
*Ampd1*‐gene: adenosine monophosphate deaminase 1
AP: action potential
*Bcl9l*‐ gene: β‐catenin transcriptional co‐activator with BCL9,
Btn: gene, *Buttonless*
*CACNA1S‐*gene: calcium voltage channel subunit alpha 1S
*Calca*‐ gene: calcitonin‐related polypeptide alpha
CGRP: calcitonin gene‐related peptide
CIP: congenital insensitivity to pain
DAPI: 4′,6‐diamidino‐2‐phenylindole
DEG: differentially expressed genes
DMEM: dulbecco’s modified eagle’s medium
DRG: dorsal root ganglia
ECL: enhanced chemiluminescence
ECS: extracellular solution
G_max_
: maximal conductance density
GO: gene ontology
GOrilla: ontology enRIchment anaLysis and visuaLizAtion tool
GRC: genome reference consortium
*Hoxc11*, *d10 and d11*‐*genes*
: homeobox C11, D10 and D11
HRP: horseradish peroxidase
IB4: isolectin B4
IOD: integrated optical density
MEOX2: mesenchyme homeobox protein 2
*Mettl2l0*‐gene: methyltransferase
MIM: mendelian inheritance in man
MrgprA3: mas‐related G‐protein coupled receptor member A3
*Mybpc1*
: 2*Mtbpc2‐* genes, myosin binding protein C1 and C2
*Myf5‐ gene*
: myogenic factor 5
*Myh1*
: *2‐* genes, myosin heavy chain 1 and 2
*Mylk2*‐gene: myosin light chain kinase
*Myom2*‐gene: myomesin 2
NGF: nerve growth factor
NGS: next generation sequencing
*NPY*‐ gene: neuropeptide Y
*Ntrk1*
: gene, Neurotrophic receptor tyrosine kinase 1
OS: overshoot
*Pax3‐ gene*
: paired box 3
*PENK*‐ gene: proenkefalin
PFA: paraformaldehyde
PGP9.5: protein gene product 9.5
PKC_Ɣ_
: protein kinase C gamma
PRDM12‐ PR: (PRDI‐BF1 and RIZ homology) domain containing member 12
ROD: relative optical densities
RT‐qPCR: real time‐quantitative polymerase chain reaction
*Ryr1*‐gene: ryanodine receptor 1
*Scn9a, 11a‐genes*
: sodium voltage‐gated channel alpha subunit 9a and 11a
SEM: standard error of the mean
TEA: tetraethylammonium
*Tnni2*‐gene: troponin I2
TrkA: ttropomyosin receptor kinase A
TRPV1: A1 or M8‐ transient receptor potential V1, A1 or M8
*Uts2b*‐ gene: urotensin 2B
VBC: vienna biocenter
VBCCF: vienna biocenter core facilities
Vmem: resting membrane potential
WT: wild‐type

## Introduction

Mesenchyme homeobox protein 2 (*MEOX2,* also known as growth arrest specific homeobox protein, Gax or *MOX‐2*) gene encodes for the transcription factor MEOX2, which is expressed in the paraxial mesoderm as early as E8.5‐9 and is critical for mammalian muscle and bone development as well as vascular differentiation [1, 2, 3]. By regulating myogenic genes such as *Pax3* and *Myf5*, it plays a role in development of limb musculature [4]. *Meox2^−/−^* (knock‐out, KO) mice are either embryonically lethal or die before weaning, have severe limb defects, a cleft palate, reduction of overall skeletal muscle mass and/or complete absence of certain muscles [4, 5]. Reduction of *MEOX2* expression has been shown in brains of Alzheimer’s disease patients [6], and its haploinsufficiency was reported to lead to neurovascular dysfunction and susceptibility to Amyloid‐β toxicity in *Meox2* ^+/−^ mice [7]. *MEOX2* mRNA dysregulation was reported in fibroblasts derived from Congenital Insensitivity to Pain (CIP) patients from two unrelated families with mutations D31Y and E172D in the transcription factor PR (PRDI‐BF1 and RIZ homology) domain containing member 12 (PRDM12) [8]. CIP is a rare genetic disorder affecting the survival or function of nociceptors, a specialised set of sensory neurons that detect pain, rendering patients unable to feel painful or noxious stimuli [9]. In the fly model, *Drosophila melanogaster* sensory neuron specific ablation of *buttonless* (*btn,* the fly homologue of *MEOX2)* results in the reduction of nocifensive behaviour in fly larvae in response to noxious heat stimulation. This suggests an important role of MEOX2 in sensory neuron function pertaining to pain [8]. However, mechanistic insight into the role of MEOX2 in the function of the mammalian sensory nervous system is unavailable.

Nociceptors are primary afferent neurons whose somata reside in dorsal root ganglia (DRG). They maintain a transcriptional programme that enables expression of specialised gene products designed for the transduction, transformation and synaptic transmission of noxious stimuli. Temperature and chemical irritant transducers expressed in nociceptors include transient receptor potential (TRP) cation channels, including capsaicin‐sensitive TRPV1 [10], and TRPA1, which is responsive to noxious heat, cold and electrophilic chemicals [11, 12] and/or heat‐sensitive voltage‐gated Na^+^ channels, for example, Na_v_1.7, Na_v_1.8 and Na_v_1.9 [13]. Nociceptor nerve endings densely innervate dermal and epidermal layers of the skin, internal surfaces and organs including gut and bone. Nociceptors integrate painful information and synapse with neurons of the central nervous system in lamina I, II and V of the spinal dorsal horn from where spinothalamic and spinoreticular tracts project to the brain.

We characterised the function of MEOX2 in sensory neurons, by analysing MEOX2‐deficient mice. In our hands, classic *Meox2* KO mice (*Meox2^−/−^*) were neonatally lethal, therefore we performed our analyses on MEOX2 haploinsufficient mutant mice, *Meox2^+/−^* (MORE, Mox‐2Cre) [5].

## Results

**Fig. 1:**
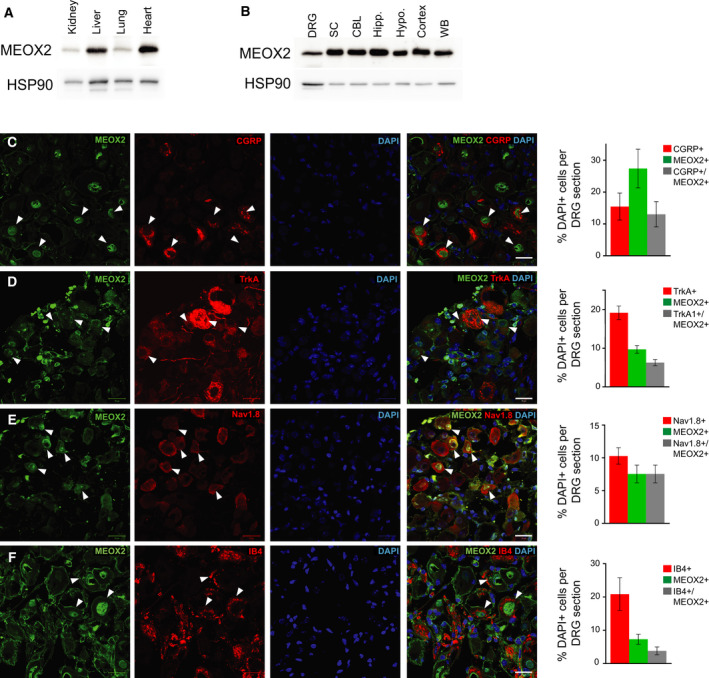
Mesenchyme homeobox protein 2 (MEOX2) is expressed in both central and peripheral nervous tissues. (A) Western blot analysis of wild‐type tissue shows relatively ubiquitous but variable expression levels of MEOX2 in kidney, liver, lung and heart, and (B) throughout the peripheral and central nervous systems; DRG – dorsal root ganglion, SC – spinal cord; CBL – cerebellum; Hipp. – hippocampus; Hypo. – hypothalamus; WB – whole brain, *n* = 1. (C) Representative sections of adult wild‐type lumbar DRG co‐immunolabelled with antibodies against MEOX2 (green) and CGRP (red); (D) TrkA (red), (E) Na_v_1.8 (red) and (F) IB4 (red). DAPI nuclear staining in blue for all panels; white arrowheads indicate double positive cells rightmost merged image for all. For CGRP, TrkA and IB4 staining, *n* = 3 WT; 2‐3 sections per 5 DRG/animal; for Nav1.8 staining *n* = 2 WT; 2 section per 5 DRG/animal. Quantification of colocalisation on the right, expressed as percentage of single or double stained DAPI+ cells. Scale bar is 20 µm.

To determine the expression of MEOX2 in wild‐type (WT) adult mouse tissue, we performed Western blotting analysis using specific antibodies against MEOX2 and noted its expression at variable levels in different tissues, such as kidney, lung and most notably liver and heart (Fig. 1A). Additionally, we noted MEOX2 to be highly expressed in both peripheral and central nervous systems, including DRG, spinal cord, cerebellum, hippocampus, hypothalamus and cortex (Fig. 1B). Immunohistochemical double labelling of DRG sections harvested from WT mice using anti‐MEOX2 antibodies together with sensory neuron markers, specifically antibodies against calcitonin gene‐related peptide (CGRP), tropomyosin receptor kinase A (TrkA), Na_v_1.8 or isolectin B4 (IB4), demonstrated that MEOX2 co‐expressed with markers for polymodal nociceptors (Fig. 1C–F). It was also present in neurons expressing the nociceptor‐specific sodium channel Na_v_1.8 (Fig. 1E) and IB4 (Fig. 1F).

**Fig. 2:**
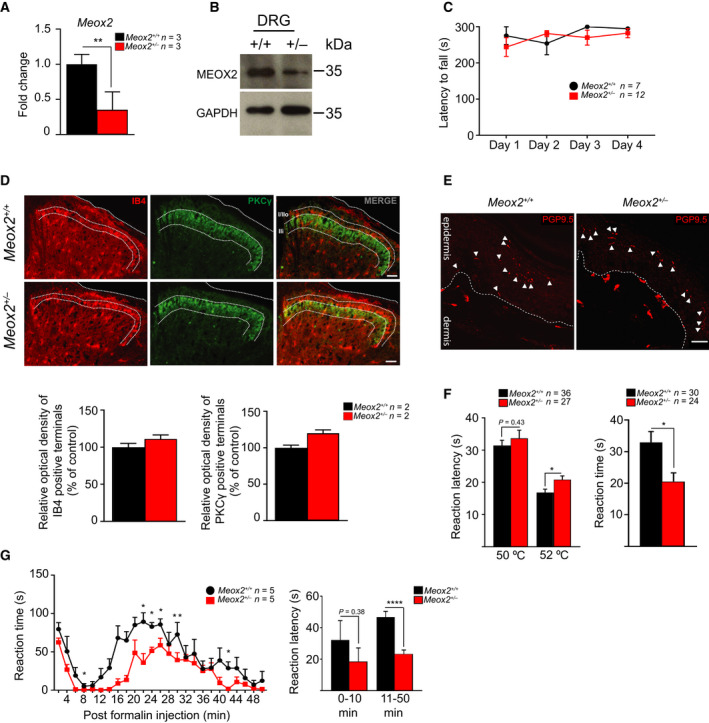
*Meox2^+/−^* mice exhibit impaired behavioural responses to acute and inflammatory pain stimuli. (A) Levels of *Meox2* mRNA in *Meox2^+/−^* (red bars) compared to *Meox2^+/+^* littermate DRG (black bars) relative to *Gapdh*. Levels of mRNA are normalised to the average of *Meox2^+/+^* samples. *n* = 3 WT and 3 HET, mean ± SEM; Unpaired Student’s *t*‐test, *P*‐value * < 0.05, ** < 0.01 or as indicated. (B) Western blot showing decreased MEOX2 expression levels in *Meox2^+/−^* DRG as compared to *Meox2^+/+^* littermate DRG. SC‐Spinal cord. GAPDH is used as a loading control. MW molecular weight marker, *n* = 1 animal per genotype. (C) No difference in the latency to fall off the accelerating Rotarod of *Meox2^+/−^* and *Meox2^+/+^* littermates; *n* = 7 WT and *n* = 12 HET, mean values ± SEM; two‐way ANOVA with Sidak’s multiple comparisons test. (D) Representative immunolabelling of the lumbar dorsal spinal cord sections of adult *Meox2^+/+^* (top row) and *Meox2^+/−^* (bottom row) littermates, with anti‐IB4 (red) and –PKCƔ (green) antibodies. Rightmost panels are merged red and green channels, with immunoreactive laminae labelled. Scale bars = 50 μm. Quantification of relative optical density (ROD), bottom, of IB4 or PKCƔ immunoreactive terminals normalised to mean of control. *n* = 2 WT and 2 HET; dorsal horns from each side (when possible) from a total of 36 spinal cord sections were counted; data represents mean ± SEM. There are no evident patterning alterations or ROD changes in the IB4 or PKCƔ labelled laminae in the *Meox2^+/−^* spinal cord sections as compared to controls. (E) Representative immunofluorescent staining of at least three animals per genotype of PGP9.5 (red) in hind paw plantar skin sections of *Meox2^+/−^* and *Meox2^+/+^* littermate control. White arrow heads indicate nerve fibres in the epidermis; scale bar 20 µm. (F) Latency to first reaction following placement on 50 °C or 52 °C hot plate (left panel); *n* = 36 WT and 27 HET; mean values ± SEM; unpaired Student’s *t*‐test; *P*‐value * ≤ 0.05; or as indicated in the figure. Right panel, reaction time (licking, biting, shaking or lifting) of hind paw following intraplantar injection of 1.0 µg capsaicin; *n* = 30 WT and 24 HET; mean values ± SEM; unpaired Student’s *t*‐test; *P*‐value * ≤ 0.05. (G) Left panel, time course of reaction (licking, biting, shaking or lifting) of hind paw following 2.5% formalin intraplantar injection. Mean reaction time ± SEM was noted every 2 min, as labelled; *n* = 5 WT and 5 HET; two‐way ANOVA with Sidak’s multiple comparisons test; *P*‐values * ≤ 0.05; ** ≤ 0.01. Right panel, average reaction time in phase I (0–10 min) and phase II (11–50 min); mean values ± SEM; unpaired Student’s *t*‐test; *P*‐value **** ≤ 0.0001, or as indicated.

To address the role of MEOX2 in nociception we analysed Meox2 haploinsufficient animals described previously [5]. We first confirmed the reduction of *Meox2* mRNA by RT‐qPCR and MEOX2 protein level by Western blot in *Meox2* ^+/−^ mice and control littermate DRG (Fig. 2A,B). Considering the critical role for MEOX2 in limb muscle development, we confirmed that *Meox2^+/−^* had no limb muscle defects by analysing their performance on an accelerating Rotarod, an assay used to test endurance, balance, grip strength and locomotion [23]. The performance of *Meox2^+/−^* on the accelerating Rotarod revealed no differences as compared to their littermate controls (Fig. 2C). We next examined the organisation of superficial spinal cord dorsal horn Rexed laminae by immunofluorescence staining of IB4 and PKC_Ɣ_, markers of laminae I and II respectively. Previous studies have implicated other homeobox containing transcription factors to organise the topographical patterning of the laminae where nociceptor projections terminate, particularly IB4‐expressing lamina I [24, 25]. However, there were no notable disruptions in the laminae I and II patterning or optical density changes of IB4 and PKC_Ɣ_ immunoreactivity in *Meox2^+/−^* spinal cord sections as compared to littermate controls (Fig. 2D). Finally, we determined that the paw skin of *Meox2^+/−^* was innervated by peripheral nociceptor endings required to detect noxious stimuli as revealed by pan‐neuronal PGP9.5 immunoreactivity (Fig. 2E). Together these data suggest that MEOX2 haploinsufficiency did not cause major hind limb muscular deficiencies, nociceptor morphological alterations or their innervation patterns in the spinal cord.

We then subjected the animals to acute heat pain by using the hot plate test. The reaction latencies of *Meox2^+/−^* mice to 50 °C were similar to their littermate controls, however, at 52 °C reaction latencies were significantly prolonged (*Meox2^+/+^* mean latency 16.84 s ± 1.049 vs. *Meox2^+/−^* 20.82 s ± 1.176; Fig. 2F, left panel), suggesting higher pain thresholds to noxious heat stimuli than their WT counterparts. Hind‐paw intraplantar injection of TRPV1 agonist, capsaicin, evoked significantly shorter reaction times in *Meox2^+/−^* animals as measured by licking, biting, shaking and lifting of the injected paw (*Meox2^+/+^* mean reaction time 32.92 s ± 3.43 vs. *Meox2^+/−^* 20.5 s ± 2.76; Fig. 2F, right panel), confirming a higher pain tolerance when compared to littermate controls. Finally, we performed the formalin test to invoke inflammatory pain by intraplantar hind paw injection of 2.5% formalin, and observing the reaction of the affected paw for 50 min [26]. The two main phases of the formalin test, phase I (0–10 min) and phase II (11–50 min), reflect acute pain responses associated with nociceptor activation in phase I, and inflammatory pain processes as well as spinal sensitisation during phase II [26, 27]. Pain‐related behaviour in response to formalin was significantly less pronounced in phase II in *Meox2^+/−^* animals as compared to littermate controls (Fig. 2G).

**Fig. 3:**
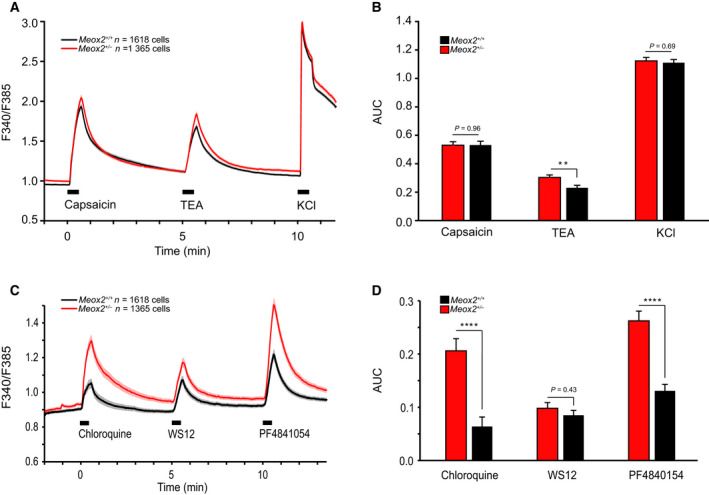
Calcium imaging responses as measured by ratiometric fluorescence intensity upon stimulation of *Meox2^+/−^* cultured sensory neurons. (A) Calcium imaging fluorescence intensity traces upon 30 s stimulation with 1 µmol·L^−1^ capsaicin, 3 mmol·L^−1^ TEA and 60 mmol·L^−1^ KCl of 1365 *Meox2^+/−^* (red trace) and 1618 *Meox2^+/+^* (black trace) neurons. Traces represent F340/F385 nm ratio of the corresponding stimulation wavelengths of Fura‐2 dye. (B) Area under the curve measured for the duration of the stimuli were similar in *Meox2^+/−^* and *Meox2^+/+^* cultured neurons. (C) Time course of cytosolic calcium upon 30 s stimulation with 100 µmol·L^−1^ chloroquine, 500 nmol·L^−1^ WS12 and 1 µmol·L^−1^ PF4941054 of 1365 *Meox2^+/−^* (red trace) and 1618 *Meox2^+/+^* neurons (black trace). Traces represent the F340/F385 nm ratio of Fura‐2 loaded neurons. (D) Area under the curve measured for the duration of the stimulation for corresponding traces from C. *n* = 2 WT and 2 HET with at least six technical replicates; data are mean ± SEM; unpaired Student’s *t*‐test; *P*‐value ** ≤ 0.01; **** ≤ 0.0001, or as indicated.

To address the observed behavioural deficiencies in MEOX2 haploinsufficiency on a cellular level, we performed intracellular Ca^2+^ measurements in cultured DRG neurons that were stimulated with specific nociceptor agonists, including TRPV1 agonist capsaicin, MrgprA3 agonist chloroquine, TRPA1 agonist PF‐4840154 and TRPM8 agonist WS12. The capacity of sensory neurons to respond to the respective chemical stimuli was measured as ratiometric fluorescence signals of the Fura‐2 Ca^2+^ indicator dye in 1365 *Meox2^+/−^* cultured DRG neurons and 1618 *Meox2^+/+^* littermate control neurons. Cytoplasmatic Ca^2+^ transients upon stimulation were similar in both genotypes for capsaicin and with KCl as a positive control; however, voltage‐gated potassium channel blocker tetraethylammonium (TEA) slightly increased Ca^2+^ transients in *Meox2^+/−^* as compared to controls (Fig. 3A,B). This indicated no impairment of capsaicin responses in *Meox2^+/−^* cultured neurons. Interestingly, we found substantially increased responses to MrgrprA3 agonist chloroquine and TRPA1 agonist PF‐4840154 in *Meox2^+/−^* cultured neurons compared to littermate control cells (Fig. 3C,D).

**Fig. 4:**
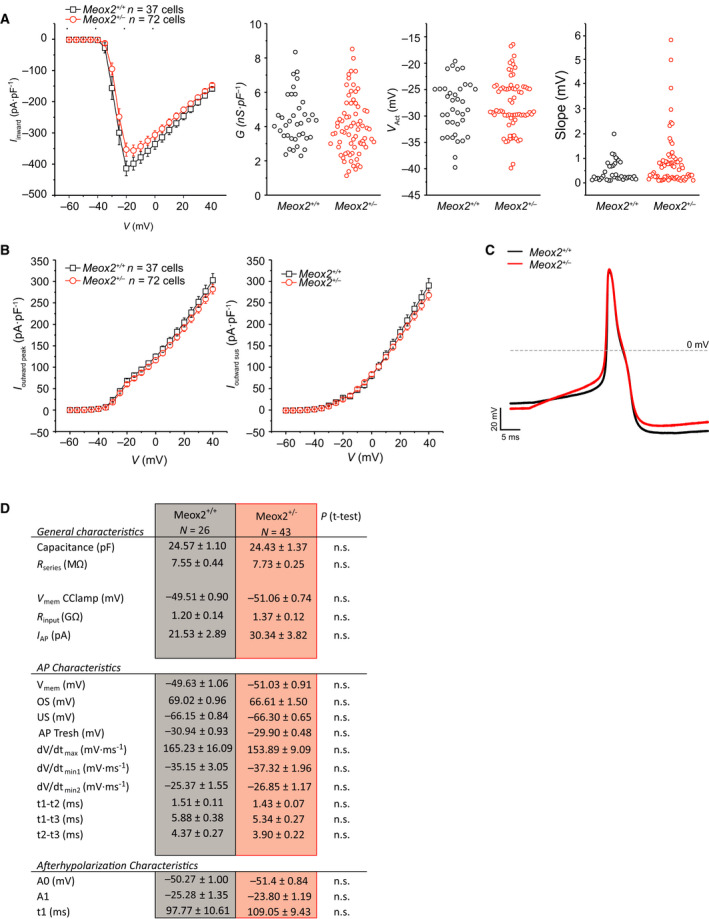
Summary of the electrophysiological characteristics of cultured *Meox2^+/−^* and littermate control dorsal root ganglia (DRG) cultures. (A) Voltage‐gated currents evoked by 5 mV depolarising pulses of Meox2*^+/−^* DRG cultures (*n* = 72 cells) and littermate controls (*n* = 37 cells) showing inward peak currents, left panel and parameters derived from inward peak currents, right panels (maximal conductance density (G), activation voltage (V_act_) and slope of activation, as labelled), reveal no difference was observed between phenotypes. (B) Outward peak voltage‐gated currents evoked by 5 mV, left panel, and outward sustained current, right panel of *Meox2^+/−^* DRG cultures (*n* = 72 cells) and littermate controls again (*n* = 37 cells), show no difference. For all panels, *Meox2^+/+^* black and *Meox2^+/−^* red traces, as labelled. (C) Representative evoked action potential by current injection recorded from *Meox2^+/−^* derived sensory neurons (red trace) compared to action potentials derived from their corresponding littermate controls (black trace). (D) Table of evoked action potential characteristics. Capacitance of the recorded DRG, reflecting the amount of plasma membrane (PM); R_series_: permeability of the PM in voltage clamp at −60 mV; V_mem_ CClamp: resting membrane potential of non‐firing DRG in current clamp recording; R_input_: input resistance, permeability of the PM in current clamp; I_AP_: minimal current injection (50ms duration) to evoke a single action potential; V_mem_: resting membrane potential during I_AP_ recordings, prior to the current injection; OS: overshoot of the action potential, maximal voltage recorded; US: undershoot of the action potential, minimal voltage recorded. AP Thres: threshold, minimal membrane voltage at which an action potential will/can be generated; dV/dt_max_: maximal speed of depolarisation; dV/dt_min1_: maximal repolarisation speed in the first phase of repolarisation; dV/dt_min2_: maximal repolarisation speed in the second phase of repolarisation; t1–t2: time in ms between dV/dt_max_ and dV/dt_min1_; t1–t3: time in ms between dV/dt_max_ and dV/dt_min2_; t2–t3: time in ms between dV/dt_min1_ and dV/dt_min2_. The afterhyperpolarisation, is fitted with a single exponential function where t1 describes the speed of recovery exp. A0 + A1(1‐(‐t/exp^t1)). DRG cultures were harvested from two WT and four HET animals.

**Fig. 5:**
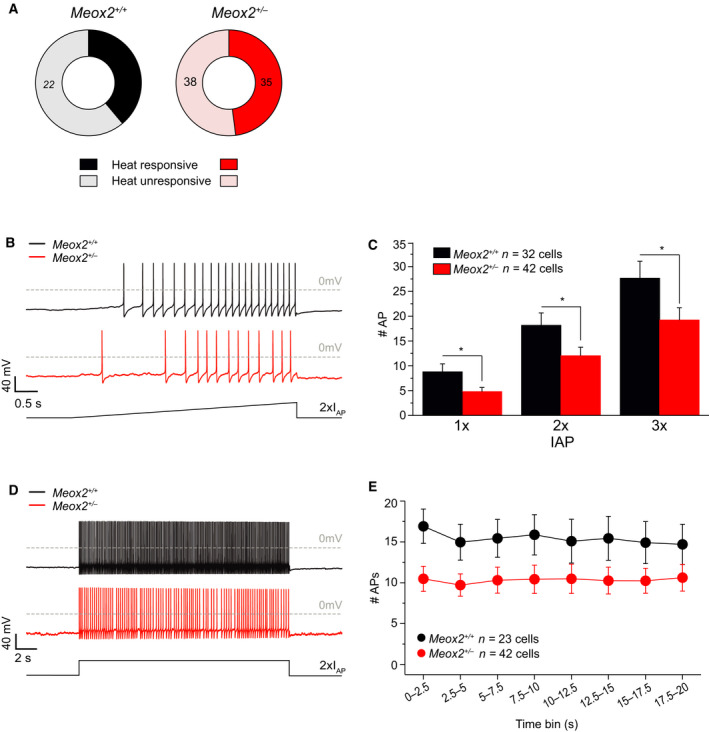
Electrical properties and heat‐responsiveness of cultured sensory neurons derived from *Meox2^+/−^* mice. (A) The ratio of heat‐responsive and non‐responsive neurons in *Meox2^+/−^* cultured dorsal root ganglia (DRG) was not different compared to their corresponding littermate controls; number of cells shown in figure; Chi‐square test, *P* = 0.37. (B) Representative traces of action potential generation upon a slowly increasing depolarising pulse in *Meox2^+/−^* and *Meox2^+/+^* DRG neurons (2x I_AP_, red and black trace respectively). (C) Quantification of the number of APs generated upon 1x, 2x and 3x IAP in *Meox2^+/−^* DRG neurons (*n* = 42) and their corresponding littermate controls (*n* = 23). (D) *Meox2^+/−^* cultured DRG (red trace) generated significantly less action potentials (#APs) compared to wildtype littermate sensory neurons (black trace) upon prolonged depolarisation. (E) The mean APs generated in 2.5 s time bins in *Meox2^+/−^* (*n* = 42) is significantly reduced compared to control littermates (*n* = 23); DRG cultures were harvested from two WT and four HET animals; mean ± SEM, *P*‐value *< 0.05, two‐way ANOVA with *post hoc* Tukey.

To determine if the behavioural phenotype in *Meox2^+/−^* animals translates to observable electrophysiological properties changes, we examined the role of MEOX2 in excitability of cultured sensory neurons from *Meox2^+/−^* mice and littermate controls. First, analysis of voltage‐gated currents evoked by 5 mV depolarising pulses, revealed no significant changes in either outward or inward currents (Fig. 4A,B). Additionally, when individual IV‐plots were modelled to determine maximal conductance density (*G*_max_), activation voltage and slope of activation, no difference was observed between phenotypes (Fig. 4A). Action potential characteristics of sensory neurons derived from *Meox2^+/−^* and their littermate controls were indistinguishable (Fig. 4C,D). Sensory neurons were challenged with a thermal stimulus to determine whether they responded with a heat‐activated current. The distribution of heat‐responsive sensory neurons was similar in *Meox2^+/−^* and littermate control neurons (Fig. 5A). However, the number of action potentials evoked by slow depolarisation was reduced in *Meox2^+/−^* sensory neurons compared to controls (Fig. 5B). Ramp‐shaped depolarisation to 1‐, 2‐ and 3‐ times the individual I_AP_, revealed a significantly reduced number of action potentials in *Meox2^+/−^* neurons (Fig. 5C). Additionally, prolonged membrane depolarisation (20 s) also showed a significantly decreased number of generated action potentials in *Meox2^+/−^* sensory neurons (Fig. 5D,E).

**Fig. 6:**
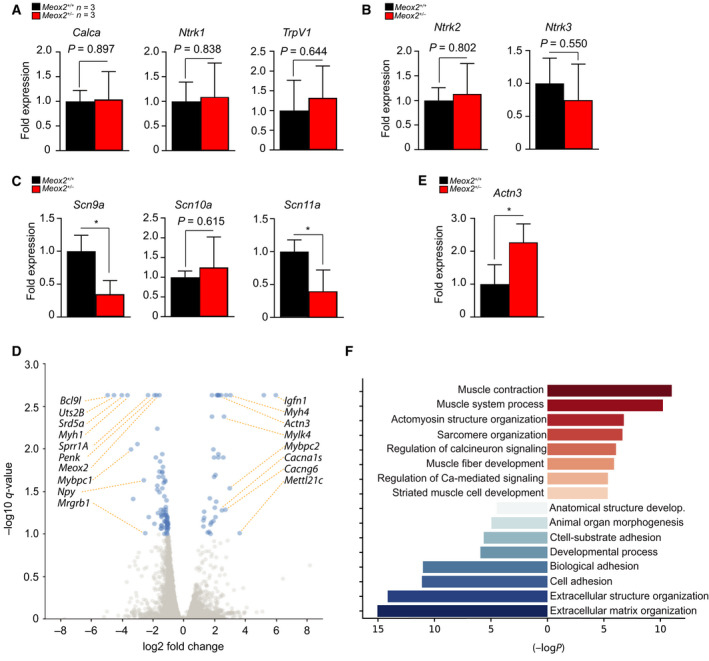
Mesenchyme homeobox protein 2 (MEOX2) deficiency in the dorsal root ganglia (DRG) causes deregulation of a number of known pain modulator and sensory neuron developmental genes. (A) Levels of mRNA in *Meox2^+/−^* (red bars) DRG for nociceptor markers *Calca*, *Ntrk1* and *TrpV1* compared to *Meox2^+/+^* littermate DRG controls (black bars) relative to *Gapdh*. Levels of mRNA for all RT‐qPCR experiments are normalised to an average of *Meox2^+/+^* samples. (B) Levels of *Ntrk2* and *Ntrk3* mRNA in *Meox2^+/−^* (red bars) compared to *Meox2^+/+^* littermate DRG controls (black bars) relative to *Gapdh*. (C) Levels of mRNA in *Meox2^+/−^* (red bars) DRG for nociceptor‐specific voltage‐gated sodium channels *Scn9a*, *Scn10a* and *Scn11a* compared to *Meox2^+/+^* littermate DRG controls (black bars) normalised to *Gapdh*. (D) Volcano plot of differentially regulated genes depicts significantly up‐ and down‐regulated genes in pooled DRG isolated from three adult *Meox2^+/−^* animals as compared to their control littermates, *n* = 3. X‐axis represents log_2_ fold change and the y‐axis represents –log_10_ (q‐values). Genes with q‐value (FDR) of less than 0.1 were assigned differentially regulated, and coloured in blue. Top pain‐ or itch‐related genes are labelled. (E) Levels of *Actn3* mRNA in *Meox2^+/−^* (red bars) compared to *Meox2^+/+^* littermate DRG controls (black bars) normalised to *Gapdh*, as one of the top upregulated hits in the transcriptome of *Meox2^+/−^* DRG. For all RT‐qPCR experiments three littermate animals per genotype were used; mean ± SEM; Unpaired Student’s *t*‐test, *P*‐values * < 0.05, or as indicated. (F) The top up‐ and down‐regulated gene ontology (GO) terms for cellular process. Enriched GO terms were analysed using GOrilla, and those with a *P*‐value < 0.001 were considered significant. Top eight GO terms are plotted both the upregulated (right side, marked in red) and downregulated (left side, in blue) with bars representing –log_10_(*P*‐value) for particular GO term.

We next sought to determine the molecular mechanisms underlining nociceptive deficiencies noted in *Meox2^+/−^* animals. Considering that MEOX2 is a transcription factor, we reasoned that there is a dysregulation of genes maintaining nociceptor cellular identity or those regulating pain responses in the DRG. We therefore performed RT‐qPCR on total RNA isolated from DRG dissected from 8‐ to 10‐week old sex‐matched *Meox2^+/−^* and control littermates using primers for genes encoding known markers of various subtypes of nociceptors, *Calca*, *Ntrk1* and the capsaicin receptor, *Trpv1*. We did not observe any difference in these genes between DRG harvested from *Meox2^+/−^* animals or littermate controls (Fig. 6A). Moreover, we did not observe any difference in expression of *Ntrk2* or *Ntrk3*, markers of mechanoreceptors and proprioceptors respectively, indicating that there was no deficit in the other major subgroups of haploinsufficient MEOX2 sensory neurons (Fig. 6B). We hypothesised that decreased number of APs upon current injection might be a consequence of impaired AP initiation or change in expression of pace‐making channels. Since MEOX2 is ubiquitously expressed including excitable tissues such as the central nervous system (Fig. 1B), muscles and heart [28, 29, 30], we posited that a nociceptor‐specific effect of MEOX2 haploinsufficiency might originate from altered expression of nociceptor‐specific ion channels. Therefore, among numerous channels affecting action potential initiation and frequency in DRG neurons, we focused on voltage‐gated sodium channels Na_v_1.7 (*Scn9a*), Na_v_1.8 (*Scn10a*) and Na_v_1.9 (*Scn11a*) all of which are preferentially expressed by nociceptors [31, 32]. While RT‐qPCR revealed no difference in expression levels of *Scn10a* mRNA between *Meox2^+/−^* and *Meox2^+/+^* DRG, two candidates with specific importance in nociceptors, *Scn9a* and *Scn11a* mRNA, were significantly downregulated in *Meox2^+/−^* (Fig. 6C).

To identify genes dysregulated in MEOX2 deficiency that additionally may modulate the reduction in painful responses to noxious stimuli we performed RNAseq on DRG dissected from either adult *Meox2^+/−^* or control littermate mice. RNAseq results are summarised in a volcano plot of log_2_ fold change vs. –log 10 (q‐value), where differentially expressed genes (DEGs) were considered significantly changed if they had a q‐value smaller than 0.1 (Fig. 6D). Levels of *Meox2^+/−^* mRNA were 0.3‐fold that of controls (q value 0.002) validating our assay and analysis. As a validation of the transcriptome DEGs, we measured the amount of *Actn3* mRNA levels by qPCR finding an increase in *Actn3* levels of 2.26‐fold in *Meox2^+/−^* samples (Fig. 6E) while transcriptome data show a 5.4‐fold increase in *Meox2^+/−^* DRG compared to the controls. Additionally, nine genes were downregulated and 17 genes upregulated ≥ 4 fold of controls (Table 1). Gene ontology (GO) enrichment analysis for cellular process using Gene Ontology enRIchment anaLysis and visuaLizAtion tool (GOrilla, http://cbl‐gorilla.cs.technion.ac.il/) revealed that the largest portion of upregulated genes were associated with terms related to function of the muscles like ‘muscle contraction’, ‘sarcomere organisation’ and ‘muscle fibre development’ (Fig. 6F). Downregulated in *Meox2^+/−^* DRGs were those genes associated with GO terms ‘extracellular matrix organisation’ and ‘biological adhesion’ as well as ‘animal organ morphogenesis’ (Fig. 6F). Intriguingly, a large portion of these gene products participate in development, cellular specification, pain perception modulation and/or sensory neuronal function. The top downregulated gene was *Bcl9l*, β‐catenin transcriptional co‐activator with BCL9, whose mRNA abundance showed a 29.9‐fold downregulation compared to control values (q‐value 0.002). BCL9/BCL9L transcriptional activation by β‐catenin is critical in sensory neurogenesis, proliferation and fate specification [33, 34, 35, 36]. *Hoxc11* (9.8 fold downregulation, q‐value 0.039), *Hoxd10* and *Hoxd11* (5.9‐fold downregulation, q‐value 0.023) are expressed in the posterior neural tube, dorsal root ganglion and hind limbs, are essential for spinal cord patterning and sensory nervous system [37, 38, 39], and were significantly downregulated in *Meox2^+/−^* adult DRG. Intriguingly, we uncovered a number of DEGs in *Meox2^+/−^* DRG which are already associated with pain perception or peripheral nerve injury, such as *Uts2b* [40], *Mybpc1* and *Mtbpc2* [41, 42], *Npy* [43] and *Penk* [47].

**Table 1:** Transcriptome analysis of *Meox2^+/−^* compared to *Meox2* ^+/+^ DRGs with top hits (q‐value < 0.1, │log_2_ fold change│> 2)

Upregulated gene	q‐value	log_2_ fold change	Downregulated gene	q‐value	log_2_ fold change
*Bcl9l*	0.002326	−4.94104	*Igfn1*	0.002326	5.97697
*Uts2b*	0.002326	−4.52092	*Lonrf3*	0.002326	5.17544
*Srd5a2*	0.002326	−4.0028	*Myh4*	0.002326	3.03753
*Myh1*	0.002326	−3.67681	*Mybpc2*	0.028767	2.97938
*Mybpc1*	0.010192	−3.42159	*Mylk2*	0.002326	2.73385
*Hoxc11*	0.038627	−3.30646	*Mylk4*	0.004157	2.60787
*Myh2*	0.00881	−3.00267	*1110002E22Rik*	0.0127	2.5692
*Hoxd10*	0.023293	−2.58451	*Actn3*	0.002326	2.43554
*Sppr1*	0.002326	−2.32377	*Cmya5*	0.002326	2.29361
			*Fsd2*	0.011493	2.26576
			*Ryr1*	0.002326	2.26065
			*Ckm*	0.0127	2.23488
			*Slpi*	0.002326	2.19309
			*Mylpf*	0.002326	2.14749
			*Ampd1*	0.041505	2.12606
			*Myoz1*	0.020283	2.04978
			*Tmod4*	0.048909	2.01775

## Discussion

Here we report that homeobox transcription factor MEOX2 is expressed in the DRG with specific markers of a subtype of sensory neurons involved in nociception. MEOX2 haploinsufficiency led to acute and inflammatory pain response deficiency in *Meox2^+/−^* murine model. While we noted a significant reduction in behavioural responses to nociceptive challenges such as noxious heat and capsaicin injections, no corresponding deficiencies in Ca^2+^ influx upon pharmacological stimulations with capsaicin, nor a decrease in capsaicin receptor, TRPV1, mRNA expression were detected. However, we did find decreased expression of *Scn9a* and *Scn11a* mRNA in *Meox2^+/−^* DRG, and these are likely to be causally involved in the decreased number of action potentials fired upon ramp current injection, thus providing a mechanistic explanation of the nociceptive behavioural phenotype.

The highly conserved family of homeobox genes were originally discovered to regulate tissue patterning in *Drosophila* [48]. Subsequently, they also participate in early embryonic tissue patterning in vertebrates and maintenance of important functions in adults [49]. In the nervous system, *Hox* genes are best known for encoding cellular and positional identity along the rostro‐caudal axis [50]. Large number of *Hox* genes are specifically expressed in the DRG [51] and *Hoxd1* and *Hoxb8* play a role in pain perception. However, loss of *Hoxb8* does not affect nociceptor function directly, but alters spinal cord patterning in laminae I and II [24, 25]. Conversely, in *Meox2^+/−^* we did not detect significant alteration of the IB4 and PKC_Ɣ_ positive laminae of the spinal cord as reported for the loss of *Hoxb8* [24]. Nevertheless, MEOX2 is expressed throughout the spinal cord and central nervous system. Therefore, we cannot rule out possible subtle alteration of the dorsal horn patterning we have not detected by immunofluorescence, or the possible unknown contribution of CNS‐expressed MEOX2 to pain processing.

Our data demonstrate that MEOX2 is primarily expressed in nuclei of both peptidergic (CGRP^+^) and non‐peptidergic (Na_v_1.8^+^ and IB4^+^) sensory neurons as revealed by co‐labelling immunofluorescence studies of adult mouse DRG sections. Nociceptive responses to temperature, capsaicin and formalin, chemical and inflammatory noxious stimuli respectively, were impaired, suggesting a critical role of MEOX2 in pain responses in mice. We did not detect a reduction of nociceptor marker transcripts including *Calca*, *Scn10a* and *Ntrk1* or *TrpV1* suggesting that overall nociceptor transcriptional identity was not perturbed with MEOX2 haploinsufficiency. This notion is supported by normal PGP9.5 immunoreactivity in the epidermis of the hind paw skin sections from *Meox2^+/−^* mice, together suggesting normal abundance and morphology of nociceptors. Additionally, there was no difference in the percentage of heat responsive vs. unresponsive cells in DRG cultures. Nevertheless, impaired behavioural responses to acute noxious stimuli and increased response of cultured DRG cells to MrgprA3 agonist chloroquine and TRPA1 agonist PF‐4840154, with no expression level change for *TrpV1* or *Calca* implies that a transcription profile of another subset of nociceptors may be affected by MEOX2 haploinsufficiency. In particular, ‘NP’ clusters of neurons described by Usoskin and colleagues [52] respond to heat, itch stimuli chloroquine and agonists Trpa1 even though they generally do not express TrpV1 [53, 54, 55, 56].

Treatment of primary DRG neurons with capsaicin and KCl resulted in calcium transients that were indistinguishable between *Meox2^+/−^* and *Meox2^+/+^*. Single cell electrophysiological recordings of cultured DRG neurons reveal indistinguishable traces of voltage‐gated currents evoked by depolarising voltage pulses. There were no significant differences in the ‘classical’ voltage‐gated Na^+^ or K^+^ currents, maximal conductance density (*G*_max_), activation voltage or slope of activation. In spite of slightly increased response to TEA in Ca^2+^ imaging, the maintained shape of action potential between *Meox2^+/−^* and control littermate cells implies that potassium channels involved in setting the resting membrane potential function properly. The chosen high KCl concentrations substantially depolarised cells and activated most voltage‐gated calcium channels; the similar responses of *Meox2^+/−^* and *Meox2* ^+/+^ did not indicate major functional changes in these channels. These results imply that basic transducer functions and neuronal excitability were unaffected. Nevertheless, MEOX2 deficiency resulted in a dramatic reduction of action potential firing, more specifically the number of action potentials generated in response to depolarising voltage stimuli. A decrease in specific voltage‐gated sodium channel expression, critical in proper AP generation, could be reflected by the impaired number of action potential firing and nociceptor‐specific voltage‐gated sodium channels Na_v_1.7 and Na_v_1.9. These have been previously described to be threshold channels, having a major role in amplifying subthreshold stimuli and therefore action potential initiation in DRG [57, 58, 59]. Importantly, we found that *Scn9a* and *Scn11a* mRNA expression significantly decreased, providing plausible mechanistic evidence that reduced action potential firing in *Meox2^+/−^* nociceptors, and therefore impaired nociception, stems from decreased expression of Na_v_1.7 and Na_v_1.9. Additionally, reduction of Na_v_1.7 and Na_v_1.9 expression also may explain the reduced latency of response to the hot plate heat challenge. Moreover, our observation is in line with previous reports finding that loss of function *Scn9a* leading to CIP in humans results in decreased action potential firing in patient iPSC‐derived nociceptors, especially upon ramp‐shaped depolarisation [60]. Additionally, mounting evidence involves Na_v_1.9 in complex physiology of inflammatory pain through histamine [61] as well as other inflammatory mediators induced mechanisms. This provides additional mechanistic evidence that impairment of the second phase of the formalin test in *Meox2^+/−^* behavioural assays theoretically could originate from decreased sensitivity to inflammatory pain due to downregulated expression of Na_v_1.9. RNAseq analysis also revealed downregulation in several nociceptive‐modulating genes including neuropeptides *Uts2b*, *Npy* and *Penk* involved in pain signalling and nerve injury [40, 44, 45, 46]. MEOX2‐mediated regulation of a subset of neuropeptides involved in nociception rises novel questions about additional layers of MEOX2‐guided functionality of nociceptors. These data raise an interesting possibility of the involvement of MEOX2 in the regulation of sodium channel function in other excitable tissues, especially in the conductive system of the heart.

Transcriptome analysis revealed a number of DEGs speculatively contributing to the nociceptor dysfunction. MEOX2 has been extensively studied for its role in early embryonic patterning, particularly in mesodermic tissue [2, 3]. Correspondingly, *Meox2^‐/‐^* animals lack specific limb muscles, and a significant reduction of others [4]. Hind limb development is particularly vulnerable to *Meox2* ablation. Indeed, the most significant GO term associated with upregulated genes in *Meox2^+/−^* DRG transcriptome was ‘muscle system process’, and significantly dysregulated genes include those associated with muscle function such as, *Actn3*, *Myom2*, *Ryr1*, *Tnni2*, *Mybpc1*, *Mettl2l0*, *Mylk2*, *Ampd1*, *Myh1* and *Myh2*. While we did not detect changes in Ca^2+^ influx upon general KCl stimulation, transcriptome analysis did reveal changes in the expression of some voltage‐gated calcium channels. In humans, mutations in *CACNA1S*, a gene upregulated in mouse *Meox2^+/−^* DRG encoding the L‐type voltage dependent calcium channel, cause types of rare, autosomally inherited periodic paralysis, hypokalemic periodic paralysis type 1 (MIM no. 170400) and susceptibility to thyrotoxic periodic paralysis 1 (MIM no. 188580). Likewise, *RYR1*, gene encoding a sarcoplasmic reticulum calcium channel in humans, and upregulated in *Meox2^+/−^* mouse DRG, causes several types of rare autosomally recessive or dominantly inherited neuromuscular or myopathy disorders [62], suggesting that MEOX2 transcriptional activity could be a common mechanism amongst these seemingly disparate neuro/muscular disorders. Of note, *Meox2^+/−^* mice in this study showed no apparent locomotion defect, nor gait, balance or stamina dysfunctions as analysed by the accelerating Rotarod performance, ruling out the possibility of confounding effects of musculature development and function in pain behaviour interpretation.

We originally observed a significant change in expression of *MEOX2* mRNA in fibroblasts harvested from CIP patients with mutations in a methyl transferase, *PRDM12* [8]. While we found co‐labelling of PRDM12 and MEOX2 in a small number of cells in the adult mouse DRG (not shown), we did not detect dysregulated *Meox2* mRNA levels in classical PRDM12 knock‐out mice [63] nor in specific PRDM12 DRG‐specific knock outs (*Prdm12fl/fl; Avil‐Cre^+^*) by RNAseq [64]. Likewise, we were also unable to detect any changes in MEOX2 protein levels in *Prdm12fl/fl; Avil‐Cre^+^* DRG by Western blotting (data not shown). A possible explanation for this discrepancy between human fibroblast and mouse DRG could be species or tissue differences. It is also possible that PRDM12 and MEOX2 coexpression is much more abundant early in development when PRDM12 expression is less restricted [65]. Therefore, the upstream transcriptional regulation of MEOX2 remains to be elucidated.

We conclude that MEOX2 has a previously undescribed but critically important role in the function of nervous system, by modulating nociception through Na_v_1.7 and Na_v_1.9 expression and consequential ability of action potential firing. Whether MEOX2 plays a similar role in other excitable tissues, such as the heart, or in the transition from acute to chronic pain is of great interest in determining the potential of MEOX2 possible therapeutic target.

## Materials and methods

### Animals

Both male and female mouse littermates 8–12 weeks of age on C57BL/6J background were used for most assays, with the exception of the intraplantar formalin injection assay, where only male animals were tested due to availability. Animals were housed at Institute of Molecular Biotechnology (IMBA), Vienna, Austria. The mouse facility maintained a 12‐h light/dark cycle and provided with food and water *ad libitum*. Experiments described in this study were approved by the Bundesministerium fur Wissenschaft, Forschung und Wirtschaft (BMWFW‐66.015/0011‐WF/V/3b/2017) and carried out according to EU‐directive 2010/63/EU. Genotyping was determined using primers previously published: morecre: 5’‐GCT GAT TTC TGA GGA TCT G‐3’ and morerev: 5’‐CTA CCC CGA ACT CTC CAC ATC TT‐3’ [5].

### Immunohistochemistry of DRG and spinal cord sections

Lumbar sections of spinal cord or DRG from control or *Meox2^+/−^* littermates were dissected into ice‐cold PBS, fixed in 4% PFA for 20 min and washed with PBS. Following cryopreservation in 30% sucrose, 20‐µm sections were cut on a cryostat (HM560, Microm International GmbH, Walldorf, Germany) at the Histopathology Service Facility at the Vienna Biocenter Core Facilities (VBCF), member of the Vienna Biocenter (VBC), Austria, mounted on glass slides and stored at −80 °C until needed.

Mounted DRG sections were permeabilised with 0.5% Triton‐X100 in PBS for 15 min at room temperature and incubated in Blocking buffer consisting of 10% normal goat serum, 1% BSA in 0.25% Triton‐X100 in PBS for 1 h at room temperature to eliminate non‐specific binding. Sections were then incubated with primary antibody at 4 °C overnight diluted in Blocking buffer raised against: MEOX2 1 : 50 (LSBio; LS‐B5472), CGRP 1 : 500 (Millipore, PC205L, Burlington, MA, USA), Na_v_1.8 1 : 1000 (Abcam; ab63331), TrkA 1 : 1000 (Abcam; ab76291), or isolectin GS‐IB4 1 : 1000 (Thermo Fisher Scientific; I21412, Waltham, MA, USA). Specifically for MEOX2 staining: following blocking, sections were washed briefly with 0.1% Tween‐20 in PBS, then incubated in goat F(ab) anti‐mouse IgG 1 : 2000 (Abcam; ab6668) diluted in 0.1% Tween 20 in PBS for 1 h and subsequently with primary anti MEOX2 antibody (1 : 50, LSBio:LS‐B5472) overnight. The following day, sections were washed with Washing buffer (0.01% Triton‐X100 in PBS) and incubated in appropriate secondary antibodies (Thermo Fisher Scientific) 1 : 500 dilution, nuclear stain DAPI at 0.2 µg·mL^−1^ (Roth, 6335.1) and where appropriate, IB4 antibody 1 : 1000 (Life Technologies; I21412, Carlsbad, CA, USA), diluted in Washing buffer for 1 h at room temperature. Finally, they were washed in PBS and coverslipped with a drop of ProLong Gold antifade mounting media (Invitrogen, P36934, Waltham, MA, USA).

The DRG incubated with anti‐CGRP, TrkA, or IB4 together with anti‐MEOX2 antibodies were imaged with Slide Scanner Vectra Polaris (Akoya Biosciences) at the Core Facility Imaging, Medical University Vienna. Analysis of the cells were performed with HALO (Indica Labs) with the Multiplex IHC Module to determine the single and double positive cells within the DRG slice. The programme was utilised for cell identification by segmenting the nuclei as DAPI positive structures, and cytoplasm detection using the preset mask. Cells immunopositive for CGRP, TrkA or IB4 alone or co‐expressed with MEOX2 were counted by experimenter blinded to the genotypes. Images of DRG sections incubated with anti‐Nav1.8 antibody were acquired using the Pannoramic FLASH 250 II slidescanner in 3D Mode at the Histopathology Service Facility at the Vienna Biocenter Core Facilities (VBCF), member of the Vienna Biocenter (VBC), Austria. The individual tissue sections were marked and exported via CaseViewer, the split images were then reassembled to stacks via a Fiji‐Macro and the slices with highest contrast were saved for further 2D‐analysis. For detecting the cells, a deep learning approach was utilised, performing annotation, training and final segmentation. Annotation was done using Qupath (https://qupath.github.io/) outlining the cells on a subset of images, then the training of the deep learning model was executed in StarDist (https://github.com/stardist/stardist). The final analysis was performed via a custom Fiji‐Macro and StarDist, applying the trained model on DRG section images, identifying the nuclei, expanding to the cytoplasm, and eliminating significant overlaps from further analysis. To define the outlines of the ganglion itself, the cell bodies were merged using a closing operation and holes were filled. The cell regions obtained via StarDist were used to quantify the signal in red (Nav1.8), green (Meox2) and blue (DAPI) channels as well as the area covered. Separate results were exported for inside and outside the ganglion in each tissue section. All datasets were presented as mean percentage of total cells (DAPI+) within a given DRG section.

Mounted spinal cord slices were hydrated with PBS then permeabilised with 0.5% TritonX‐100 in PBS. Non‐specific binding was blocked with 5% normal goat serum in 0.25% TritonX‐100 in PBS. Following two washes in Washing buffer, sections were blocked with 1 : 2000 dilution goat F(ab)anti mouse (IgG antibody) (Abcam, ab6668) in Washing buffer for 1 h at room temperature. Sections were incubated with primary antibody overnight at 4 °C raised against PKC_Ɣ_ at 1 : 500 dilution (Santa Cruz, sc‐211) in Blocking buffer. Following three washes in Washing buffer, sections were incubated with appropriate secondary antibodies (Thermo Fisher Scientific) in PBS with 0.1% Tween 20 and 0.01% TritonX‐100 at 1 : 500 dilution, DAPI 1 : 2000 (D1306, Invitrogen, Waltham, MA, USA) and isolectin GS‐IB4 at 1 : 500 dilution (Thermo Fisher, I21412). Following another three washes in PBS with 0.01% Tween 20 and 0.01% TritonX‐100 sections were coverslipped with DAKO Fluorescent Mounting media (Agilent Technologies, Austria, S3023) and imaged on the Pannoramic FLASH 250 II slidescanner in 3D Mode at the Histopathology Service Facility at the VBCF.

Individual tissue sections were marked and exported via SlideViewer and analysis of IB4 or PKC_Ɣ_ immunopositive terminals in the spinal dorsal horn was performed with imagej software by experimenter blinded to the genotypes. Briefly, relative optical densities (ROD) were obtained by subtracting the background integrated optical density (IOD) from the positively stained region of interest IOD, which was manually traced in each section. Data from immunofluorescence were expressed as fold change in *Meox2^+/−^* vs. control littermates. Dorsal horns from each side (when possible) from 36 spinal cord sections prepared from two animals of each genotype were analysed.

### Skin biopsies

Following sacrifice, skin biopsies were collected from hind paws, flattened and fixed in 4% PFA in PBS overnight at 4 °C. Following cryopreservation in 30% sucrose, 20 µm sections were cut at VBCF. Subsequently, sections were incubated in 50 mm glycine for 45 min at room temperature. Following two washes in 0.1% Triton‐X100 in PBS, sections were incubated in Blocking buffer containing 1% BSA, 10% normal goat serum, 0.1% Triton‐X100 in PBS, for 1 h. Sections were then incubated at 4 °C overnight, in anti‐PGP9.5 antibody (1 : 200, Zytomed; 516‐3344) in Blocking buffer. Following two washes with Washing buffer, sections were incubated in appropriate secondary antibodies diluted 1 : 500 in Washing buffer for 1 h at room temperature, washed in PBS, coverslipped with a drop of ProLong Gold antifade mounting media and imaged on laser scanning confocal microscope Zeiss LSM700.

### Western blotting

After sacrificing animals, tissue samples were collected in RIPA (Sigma‐Aldrich, St. Louis, MO, USA) buffer on ice, with Proteinase and Phosphatase Inhibitors Cocktail (Thermo Fischer Scientific), lysed and homogenised using a Precellys 24 Tissue Homogenizer (Bertin Instruments, 3 × 30 s, 5000 r.p.m.) and protein concentration measured using the Bradford assay (Bio‐Rad). Laemmli buffer (Sigma) treated samples with standardised concentration of 1 µg·µL^−1^ were incubated at 95 °C for 5 min. Per lane 25 µg of protein was loaded on 10% PAA gel, followed by protein transfer to a methanol activated PVDF membrane (GE Healthcare Life Sciences) over night at 4 °C with constant current of 0.12 A. The membrane was blocked with 5% BSA in TBS‐T (0.01% Tween 20) for 1 h at room temperature. Anti‐MEOX2 1 : 1000 (LS Bio; LS‐B5472) and GAPDH 1 : 1000 (HRP conjugate, Cell signalling; 3683S, Danvers, MA, USA) or HSP90 1 : 1000 (Cell signalling, C45G5) antibodies diluted in Blocking buffer were used for detection of MEOX2 or loading controls respectively, followed by anti‐rabbit or anti‐mouse HRP‐conjugated secondary antibodies 1 : 30 000 (Cytiva, NA934V; Sigma‐Aldrich, GENA931), if required, also diluted in Blocking buffer. The signal was visualised with a chemiluminescent reagent (ECL, GE Healthcare Life Sciences) and imaged (ChemiDoc Imaging System, Bio‐Rad).

### Behavioural assays

All pain assays were performed once on each animal, separated by at least 24 h between tests to reduce stress.

#### Hot plate assay

Briefly, the first day of testing, animals were placed on a hot plate (Ugo Basile) set for 50 °C and manually observed for first reaction. Counted reactions included: jumping, licking, shaking or lifting of the hind paws. The animal was removed from the apparatus at first reaction or when no reaction was observed within 60 s. 24 h later, animals were tested for first reaction to being placed on a 52 °C hot plate.

#### Intraplantar capsaicin or formalin injections

1 µg of capsaicin (Sigma; M2028) diluted in 15 µL PBS was injected intraplantar in the hind paw. The animal was then observed for 5 min and timed for duration of reaction. 2.5% formalin (830 mm, Thermo Fischer Scientific) was diluted in 20 µL PBS and was injected in the hind paw of the experimental animal which was then observed for 50 min and timed for the duration of reaction. Counted reactions for both tests included: licking, shaking or lifting of the injected paw. In response to the formalin test, reaction time per 2 min bins was reported. At the completion of the test, the animal was returned to its home cage.

#### Accelerating rotarod performance test

Accelerating rotarod was performed on a 5–40 r.p.m. accelerating apparatus (Ugo Basile), as described previously [14]. Briefly, mice were habituated to the rotating rotarod apparatus (Ugo Basile) once for a 1‐min trial, at 5 r.p.m. Following habituation, each mouse was given one trial per day, for four consecutive days, on the 5–40 r.p.m. accelerating rod.

### Sensory neuron culture

Lumbar DRG were harvested from adult mice as previously published [15, 16]. After removal of the connective tissue from the ganglia, they were incubated twice in Liberase (90 mg·L^−1^ DMEM) for 30 min. After washing with PBS, the tissue was incubated with 0.05% Trypsin‐EDTA for 15 min and subsequently washed with TNB medium (Biochrom, Merck Millipore, Berlington, MA, USA) supplemented with l‐glutamin (0.2 mm), penicillin and streptomycin (200 U·mL^−1^), and protein–lipid complex (Biochrom, Merck Millipore). The DRG were dissociated with a fire‐polished Pasteur pipette and centrifuged through a 3.5% BSA gradient to eliminate debris and non‐neuronal cells. The pelleted sensory neurons were resuspended, plated on coverslips coated with poly‐l‐lysine/laminin‐1 (Sigma‐Aldrich), and cultivated in supplemented TNB containing mNGF 2.5 s (25 ng·mL^−1^) at 37 °C and 5% CO_2_ in a humidified incubator.

### Single cell electrophysiology

Cultured sensory neurons were used for electrophysiological experiments 16–24 h after seeding. Glass coverslips were mounted in a recording chamber and placed on a Zeiss Axiovert 200 microscope. All measurements were recorded with an EPC 10 and the patchmaster v2.73 software (HEKA) at room temperature. From isolated sensory neurons, membrane potential was recorded in whole‐cell current‐clamp configuration of the patch clamp technique in extracellular solution (ECS) containing (in mmol·L^−1^); NaCl (150), KCl (5), CaCl_2_ (2), MgCl_2_ (1), HEPES (10), Glucose (10) and the pH was set to 7.3 with NaOH. Borosilicate glass pipettes (Science Products) were pulled with a horizontal puller (P‐97, Sutter Instruments Company) and filled with an intracellular solution composed of (in mmol·L^−1^); K gluconate (98), KCl (50), CaCl_2_ (0.5), MgCl_2_ (2), EGTA (5), HEPES (10), MgATP (2), NaGTP (0.2) and pH adjusted to 7.3 with KOH. The recorded neurons were held at 0 pA. The minimal current to evoke a single action potential within 50 ms (I_AP_) was obtained by 5 pA increasing depolarising pulses. For the slow depolarisation (5 s) of the membrane, current injections where applied from 0 pA to 1×, 2× and 3× I_AP_ of the respective neuron and amplitude of the 20 s depolarising pulse was set to 2× I_AP_ [16].

A seven‐barrel application system (Dittel, Prague) with common outlet was used for heat stimulation of single neurons [17]. Heat‐activated inward currents (I_Heat_) were elicited by applying ramp‐shaped heat stimuli (linear temperature increase from room temperature to 50 °C within 5 s).

The input resistance of sensory neurons was determined by four‐increasing hyperpolarising current injection steps (Δ − 5 pA per step from a holding current of 0 pA, 5 kHz). The average resistance was calculated according to Ohm’s law. From the AP evoked by injecting depolarising current pulses (50 ms, sampled at 20 kHz), the resting membrane potential (Vmem), afterhyperpolarisation and overshoot (OS) as well as the rheobase in *Meox2^+/+^* and *Meox2^+/−^* DRG neurons were determined. From the first derivative of the evoked APs, the maximal speed of depolarisation and the biphasic repolarisation were derived. The AP threshold was determined as the turning point of the first derivative and the according membrane voltage was deducted [15, 16, 18].

### Microfluorimentric Ca^2+^ measurements

Sensory neuron cultures for calcium imaging were prepared similarly to the method described above, with the difference that DRG from all spinal levels were used, from two *Meox2^+/+^* and two *Meox2^+/−^* adult 18 weeks old male mice. DRG were digested in Dulbecco’s modified Eagle’s medium (DMEM) containing 1% streptomycin/penicillin, treated with 1 mg·mL^−1^ collagenase (Sigma‐Aldrich) and 3 mg·mL^−1^ Dispase II (Roche Diagnostics) for 60 min, mechanically dissociated with a Pasteur pipette and plated onto 12 mm glass coverslips previously coated with 100 µg·mL^−1^ poly‐D‐lysine (Sigma‐Aldrich). DRG neurons were cultured in DMEM culture medium supplemented with 10% BSA and 100 µg·mL^−1^ streptomycin/penicillin (all from Biochrom, Berlin, Germany) 2% B‐27 Supplement (50×, serum free, Gibco; 17504044). Neurons were cultured at 37 °C and 5% CO_2_ for 15–30 h and loaded with Fura‐2 AM (3 µm, 30 min at 37 °C, containing also 0.02% Pluronic, both from Life Technologies GmbH, Darmstadt, Germany) and left to recover for about 10 min in ECS (as above except for a lower calcium concentration of 1.25 mmol·L^−1^ CaCl_2_), which was also used as continuous gravity‐driven superfusion in the experiments. The coverslips were placed in glass bottom dishes which were then mounted in an Olympus IX73 inverted microscope and imaged using a 10x objective. Time‐course experiments were done using a software‐controlled eight‐channel common‐outlet system (ALA Scientific Instruments Inc., Farmingdale, New York, USA) with cells permanently superfused with ECS or stimuli dissolved in ECS, as follows: chloroquine 100 µm (Sigma‐Aldrich), WS12 500 nm (Santa Cruz Biotechnologies Inc., Dallas, TX, USA), PF‐4840154 1 µm (MedChemExpress), capsaicin 1 µm (Sigma‐Aldrich), TEA 3 mm (Sigma‐Aldrich). Stimulation with an ECS containing KCl 60 mm (isoosmolar substitution of NaCl) served as a positive control. Cells were superfused with stimuli solutions for 30 s, following 270 s superfusion with ECS. Fura‐2 was alternatingly excited for 30 ms by a 340 nm (50 mWatt, used at 100%) and 30 ms by a 385 nm (1435 mWatt, used at 5%) LED using an Omicron LEDHub (Omicron‐Laserage Laserprodukte, Rodgau‐Dudenhofen, Germany). Fluorescence emission was long‐pass filtered at 495 nm and pairs of images were acquired at a rate of 1 Hz with a 4.2 Megapixel 16 bit CCD camera (6.5 µm pixel edge length, 18.8 mm sensor diameter, PRIME BSI, Teledyne Photometrics, Tucson, Arizona USA). The hardware was controlled by the µmanager 1.4 plugin in imagej. The background intensity was subtracted before calculating the ratio between the fluorescence emitted when the dye was excited at 340 nm and at 385 nm (F340/F385 nm). The time course of this ratio was analysed for regions of interest adapted to individual cells. The area under the curve of the stimulation periods was used to quantify response magnitude. The calculation uses a reference period immediately before the period of interest, the resulting unit of Ratio*time was omitted for simplicity.

### RNA sequencing

Dorsal root ganglia were isolated from 3 *Meox2^+/+^* and 3 *Meox2^+/−^* littermates (14 ± 2 weeks of age) into RNA*later* ^TM^ Stabilisation Solution (Thermo Fisher) and frozen at −80 °C. Tissues were homogenised in buffer RLT using a TissueLyser II (Qiagen, Hilden, Germany 10 × 30 s, 30 Hz) before centrifugation through QIAshredder spin columns (Qiagen). RNA was isolated using an RNeasy Mini Kit (Qiagen), following the manufacturer’s instructions. The amount of RNA was quantified using a Qubit 2.0 Fluorometric Quantitation system (Life Technologies) and an Experion Automated Electrophoresis System (Bio‐Rad) was used to calculate RNA integrity score. RNA‐seq libraries were prepared with the TruSeq Stranded mRNA LT sample preparation kit (Illumina) using Sciclone and Zephyr liquid handling robotics (PerkinElmer) at pre‐ and post‐PCR steps. Library concentrations were determined using a Qubit 2.0 Fluorometric Quantitation system (Life Technologies) and the distribution of sizes determined using an Experion Automated Electrophoresis System (Bio‐Rad). To sequence, the nine libraries were pooled, diluted to equimolar amounts and sequenced on an Illumina HiSeq 3000/4000 using 50 bp single‐end chemistry. Base calls provided by the real‐time analysis software (Illumina), were converted into multiplexed, unaligned BAM format before demultiplexing into sample‐specific unaligned BAM files. Custom programs based on Picard tools (https://broadinstitute.github.io/picard/) were used for raw data processing.

### Transcriptome analysis

Transcriptome analysis was performed with the Tuxedo suite. Per sample, NGS samples (passing vendor quality filtering) were aligned to the UCSC Genome Browser (hg38/mm10) flavour of the Genome Reference Consortium (GRCh38/GRCm38) assembly with TopHat2 (v2.1.1) [19], a splice junction mapper utilising the Bowtie2 (v2.2.9) [20] short read aligner. Basic Ensembl transcript annotation from version e87 (December 2016) served as a reference transcriptome. Cufflinks (v2.2.1) [21] was used for transcriptome assembly, on the basis of spliced read alignments and the reference transcriptome, as well as raw transcript quantification. Transcriptome sets of each sample of each group were combined using the Cuffmerge algorithm, then the differential expression calling was done using Cuffdiff [22]. The cummerbund and biomaRt Bioconductor packages (www.bioconductor.org/packages/release/bioc/html/cummeRbund.html and www.bioconductor.org/packages/release/bioc/html/biomaRt.html) were utilised in custom R scripts to perform quality assessment and further refinement of analysis results.

### Real time‐qPCR

The DRG from three *Meox2^+/+^* and three *Meox2^+/−^* mice were isolated into TRIzol Reagent (Thermo Fisher). Tissues were lysed and homogenised using a Precellys 24 Tissue Homogenizer (Bertin Instruments, 3 × 15 s, 5000 r.p.m.) the RNA isolated, treated with PerfeCTa DNase I (Quantabio) and reverse‐transcribed using iScript cDNA Synthesis Kit (Bio‐Rad), following the manufacturer’s instructions. cDNA amounts were normalised after quantification on a Nanodrop^TM^ 2000 Spectrophotometer. RT‐qPCR was performed using an iTaq Universal SYBR Green Supermix (Bio Rad) on a StepOnePlus Real‐Time PCR System (Applied Biosystems) with primers as listed in Table 2. The comparative Ct method (ΔΔCt) was used to determine relative gene expression, normalising to *Gapdh*.

**Table 2:** List of primers used for RT‐qPCR

Gene	Protein product	Forward primer (5‘→3‘)	Reverse primer (5‘→3‘)
*Gapdh*	GAPDH	GGTCGTATTGGGCGCCTGGTCACC	CACACCCATGACGAACATGGGGGC
*Meox2*	MEOX2	GCTGATTTCTGAGGAGGATCTG	CTACCCCGAACTCTCCACATCTT
*Cacna*	CGRP	GCAGTGCCTTTGAGGTCAATC	GTGACACTAGAGCCCTCAGC
*Ntrk1*	TrkA	AACAACGGGAACTACACCCT	TGTGCTGTTACCGTCCACT
*Ntrk2*	TrkB	GATCCTGGTGGCTGTGAAGAC	GTGTGCCCTAAGGAACTTGTTGA
*Ntrk3*	TrkC	TACTACAGGGTGGGAGGACAC	CCGTGTTGGAAAGCTGGAAC
*Scn9a*	Nav1.7	TGGGCGAATTCACCTTCCTC	CCACGATGGTTTTTAGTCCTGG
*Scn10a*	Nav1.8	ACCGACAATCAGAGCGAGGAG	ACAGACTAGAAATGGACAGAATCACC
*Scn11a*	Nav1.9	CAATCAGCAGCAGAAAAAGTTAGG	TGGCCTTCAGATTCAGCCAT
*Kcna2*	Kv1.2	ATTGACATCGTGTCGGTGCT	CTCACGGTAGCTGTGCTTGA
*Kcnb2*	Kv2.1	CGTCATCGCCATCTCTCATG	CAGCCCACTCTCTCACTAGCAA
*Actn3*	ACTN3	GAGGGACGCACTAGAGAGGA	GCCAGTTATTGAAGGGGGCT

### Data‐analyses and statistics

All values are given as mean ± SEM. Data were analysed using origin software (Originlab) for electrophysiological experiments. Statistical analysis was performed in graphpad Prism 7.0 by unpaired Students *t*‐test, one‐way or two‐way ANOVA and Tukey or Sidak *post hoc* testing and Chi‐square test, where appropriate. Differences were considered statistically different if *P* < 0.05.

## Conflict of interest

The authors declare no conflict of interest.

## Author contributions

TK performed and analysed the calcium imaging and RNAseq experiments and contributed to the data interpretation and manuscript conceptualisation, EML performed immunohistochemical experiments and aided in behavioural experiments, ML performed and analysed the electrophysiological experiments and contributed to the data interpretation and manuscript conceptualisation, CIC and MJMF supervised the calcium imaging experiments, CWF performed and analysed the RT‐qPCR experiments, AS performed immunohistochemical experiments, MK supervised the electrophysiological experiments and edited the manuscript draft, JMP contributed to the conceptualisation of the project and provided the mouse model, VN conceptualised, designed, lead, organised and supervised the project, performed and analysed the behavioural data, interpreted the data and wrote the manuscript. All authors contributed to and edited the manuscript.

### Peer Review

The peer review history for this article is available at https://publons.com/publon/10.1111/febs.16347.

## Supporting information


Supporting information

## Data Availability

All data generated or analysed during this study are included in this published article in the form of graphs (under the Supporting information). The raw data used and/or analysed during the current study are available from the corresponding author on reasonable request.
